# Nutrition and physical activity counselling by general practitioners in Lithuania, 2000–2014

**DOI:** 10.1186/s12875-019-1022-8

**Published:** 2019-09-07

**Authors:** Vilma Kriaucioniene, Janina Petkeviciene, Asta Raskiliene

**Affiliations:** Faculty of Public Health, Academy of Medicine, Lithuanian University of Health Sciences, Tilzes 18 Kaunas, Lithuania

**Keywords:** Counselling, Nutrition, Physical activity, General practitioners, Overweight

## Abstract

**Background:**

Primary health care plays a crucial role in providing recommendations on a healthy diet and physical activity to assist patients in weight management.

The study aimed to evaluate health behaviour counselling provided by general practitioners (GPs) for adults with overweight and obesity in Lithuania between 2000 and 2014.

**Methods:**

Eight biennial postal surveys to independent nationally representative random samples of Lithuanians aged 20–64 were conducted. Response rates varied from 41.1 to 74%, with a decreasing trend over time. The data of 5867 participants who visited a GP at least once during the last year and had BMI of ≥25.0 kg/m^2^ were analysed. Respondents were asked about GP advice on nutrition and physical activity and changes in their health behaviour during the last year.

**Results:**

The proportion of persons with overweight who reported GP advice on nutrition increased from 23.6% in 2000 to 37.5% in 2010 and advice on physical activity from 11.9 to 17.2% respectively; however, later both proportions decreased slightly. The likelihood of reporting was higher in respondents with higher BMI, more chronic conditions and frequent contact with a GP. Respondents who were living in cities, older and highly educated women were all more likely to report being advised on physical activity. Men and women who received advice from a GP more often reported changes in health behaviour as compared with non-advised individuals.

**Conclusions:**

Despite increasing trends, the rate of GP advice on nutrition and physical activity reported by patients with overweight and obesity remains low in Lithuania. GP advice appears to have a significant impact on attempts by patients to change behaviour related to weight control. Therefore, there is an obvious need to make additional efforts to increase the frequency of GP counselling and to identify and address barriers to advising patients with overweight.

## Background

Overweight and obesity are associated with increased mortality worldwide [1, 2]. Obese individuals are at high risk for a range of chronic diseases, including cardiovascular diseases, diabetes, some cancers, musculoskeletal disorders, and others [3, 4]. In Lithuania, the prevalence of obesity and obesity-related disorders is one of the highest in Europe [5, 6]. Over the last two decades (1994–2014), the proportion of overweight men increased from 47.0 to 58.6%, while the proportion of overweight women slightly decreased from 51.7 to 46.0% [6]. In 2014, the prevalence of obesity was 19.5% in Lithuanian men and 17.3% in women [6].

Obesity results from a combination of individual and environmental factors such as dietary patterns, physical activity, genetics, food and physical activity environment, and food marketing. No single intervention can prevent the rise of the obesity epidemic. Activities at both population and individual levels are important [7]. Population-based interventions consist of health promotion and physical, economic and regulatory actions that influence the environment. Primary health care plays a crucial role in providing recommendations on a healthy diet and physical activity to assist patients in weight management [8]. The previous studies showed that general practitioners (GPs) were usually regarded as the most reliable sources of health information. Many people prefer to receive health recommendations from their health care practitioners than from mass media, internet, friends and other sources [9, 10]. However, a low rate of counselling on health behaviour provided by GPs for overweight people has been demonstrated [11, 12]. Limited consultation time, lack of specific education and motivation are still important barriers to providing health education in primary health care [13, 14]. Meanwhile, scientific evidence shows that education about healthy diet and physical activity has the potential to change individual health behaviour, even though counselling sessions are rare and short [15, 16].

After regaining independence in 1990, health care reform was introduced in Lithuania. The establishment of a family medicine institution was recognised as a priority of health care reform [17]. Health education and disease prevention are among the most important tasks of a new primary health care system. Several studies examined the changes in GP task profiles during the implementation of health care reform in Lithuania [17, 18]. However, surveys assessing the extent of counselling on health behaviour in primary health care are lacking.

This study aimed to evaluate nutrition and physical activity counselling provided by GPs for adults with overweight and obesity in Lithuania between 2000 and 2014.

## Methods

### Study design and sample

The data from eight cross-sectional surveys of Lithuanian Health Behaviour Monitoring were analysed. Between 2000 and 2014, the surveys have been carried out every second year. For every survey, a nationally representative simple random sample aged 20–64 years was drawn from the National Population Register [19]. The sample consisted of 3000 individuals in each of 2000–2008 survey and 4000 individuals in each of the last three surveys. The questionnaires, which have remained essentially unchanged over the study years, were mailed between April and June with one reminder [20]. Response rates were 74% in 2000, 64% in 2002, 62% in 2004, 59% in 2006, 61% in 2008, 54% in 2010, 51% in 2012 and 41% in 2014. The Lithuanian Bioethics Committee approved all surveys (protocol No. 6B-10-61). Written informed consent for participation was obtained from all respondents.

In total, 14,804 individuals (6211 men and 8593 women) participated in the surveys. Respondents were asked how often they had seen a GP during the previous 12 months. The majority of men (4353 or 70.1%) and women (7144 or 83.1%) visited a GP at least once during the last year. The prevalence of overweight was 39.3% in men and 28.1% in women; the prevalence of obesity was 18.3 and 18.9% respectively. For this study, the participants (2508 men and 3359 women) who visited a GP during the last year and were overweight or obese (body mass index (BMI) ≥25.0 kg/m^2^) were selected.

### Measurements

Self-reported current weight and height were used to calculate BMI as weight in kilograms divided by height in meters squared. According to WHO guidelines, overweight was defined as BMI ≥ 25 - < 30 kg/m^2^ and obesity as BMI ≥ 30 kg/m^2^ [7].

Information on receiving advice from a GP was obtained from the following survey questions: ‘During the last year (12 months) have you been advised by a GP to change your dietary habits?’ and ‘During the last year (12 months) have you been advised by a GP to increase your physical activity?’. Respondents were asked about changes in their health behaviour: ‘During the last year (12 months) have you changed your diet or other habits for health reasons?’ The possible changes were listed: 1) ‘I consumed less fat’, 2) ‘I have changed the type of fat I eat’, 3) ‘I consumed more vegetables’, 4) ‘I used less sugar’, 5) ‘I used less salt’, 6) ‘I increased physical activity’. The answer was ‘Yes’ or ‘No’ for every statement.

The respondents were grouped into four age groups: 20–34, 35–44, 45–54 and 55–64 years. By education, respondents were categorized into three groups: 1) secondary school or lower education, 2) college or vocational school and 3) university education. According to the administrative classification of places of residence, the respondents were grouped as living in cities (capital city and four largest cities of Lithuania) and other places (towns and villages). The addresses of respondents, obtained from the National Population Register, were used for determination of living place. Marital status was dichotomised as ‘married’ and ‘others’.

Respondents were asked about chronic diseases, such as hypertension, coronary heart disease, diabetes, chronic bronchitis or asthma, osteoporosis, and other, treated or detected by a doctor during the last year. According to the answers, they were categorized into four groups: no diseases, 1, 2 and 3 or more diseases. By the number of visits to GP during the last year, respondents were grouped as visiting 1–2 times and 3 times or more.

The characteristics of the study population are presented in Table 1. There were no substantial differences in the characteristics of respondents between study years, except higher education of women in the last surveys (data are not shown).

**Table 1:** Characteristics of the study population (%)

Characteristics	Males *n* = 2508	Females *n* = 3359	p
Age			*p* < 0.001
20–34	19.3	12.1	
35–44	25.1	19.4	
45–54	28.7	33.9	
55–64	26.9	34.6	
Highest educational attainment			*p* < 0.001
Secondary school or lower	45.7	37.7	
Vocational school or college	31.8	38.6	
University	22.5	23.7	
Place of residence			0.748
City	42.4	42.0	
Another place	57.6	58.0	
Body mass index			*p* < 0.001
≥ 25 - < 30 kg/m^2^	68.3	59.7	
≥ 30 kg/m^2^	31.7	40.3	
Family status			*p* < 0.001
Unmarried	18.2	31.3	
Married	81.8	68.7	
Number of chronic conditions (last 12 month)			*p* < 0.001
0	28.9	28.8	
1	39.6	32.0	
2	17.9	19.5	
≥ 3	13.6	19.7	
Number of the visits to a GP (last 12 month)			*p* < 0.001
1–2	53.9	37.1	
≥ 3	46.1	62.9	

*GP* general practitioner

### Statistical methods

Data analysis was performed using the statistical package IBM SPSS Statistic 20. The categorical variables were presented as percentages and compared using the chi-square test. The normal approximation was used in the calculation of 95% confidence intervals for proportions. Secular trends in the extent of counselling on health behaviour between 2000 and 2014 were tested using multivariate logistic regression analysis where study year was included as a continuous variable. The same analysis was used to evaluate the associations between reporting advice on nutrition and physical activity and characteristics of respondents, as well as reported changes in health behaviours. *P*-values of less than 0.05 were considered statistically significant.

## Results

The proportion of patients with overweight and obesity who reported being advised by a GP to change nutrition habits increased from 23.6% in 2000 to 37.5% in 2010 (*p* < 0.05) (Table 2). In the last two surveys, this proportion was slightly lower than in 2010. Between 2000 and 2008, the proportion of respondents reporting that they were recommended to increase physical activity rose from 11.9 to 18.0% (p < 0.05). A lower rate of counselling on physical activity was found in subsequent surveys.

**Table 2:** The proportion of respondents with overweight and obesity who were advised by a general practitioner to change their nutrition and physical activity habits in 2000–2014

Year	Advice to change nutrition habits		Advice to increase physical activity	
	Percentage	95% CI	Percentage	95% CI
2000 *n* = 699	23.6	20.4–26.8	11.9	9.6–14.1
2002 *n* = 680	29.1	25.7–32.5	13.8	11.3–16.3
2004 *n* = 670	26.7	23.8–30.4	11.7	9.4–14.1
2006 *n* = 705	27.1	23.8–30.4	12.7	10.3–15.1
2008 *n* = 738	33.9	30.5–37.3	18.0	15.3–20.8
2010 *n* = 883	37.5	34.3–40.7	17.2	14.9–19.6
2012 *n* = 779	31.3	28.1–34.6	15.3	12.8–17.8
2014 *n* = 713	30.0	26.9–33.4	11.6	9.2–13.9
Overall *n* = 5867	30.2	29.0–31.4	14.2	13.3–15.1

*CI* confidence interval

Multivariate logistic regression analysis showed that in men the odds of reporting advice on nutrition increased by 11% and on physical activity by 7% per each two-year study period (Table 3).

**Table 3:** Odds ratios of receiving advice from a general practitioner by analysed variables in overweight men (multivariate logistic regression analysis)

Variables	Advice on nutrition		Advice on physical activity	
	OR	95% CI	OR	95% CI
Age				
20–34	1		1	
35–44	1.3	0.96–1.89	1.4	0.93–2.21
45–54	1.3	0.95–1.83	1.1	0.71–1.68
55–64	1.4	0.97–1.91	0.9	0.63–1.51
Highest educational attainment				
Secondary school or lower	1		1	
College or Vocational school	0.9	0.69–1.01	0.9	0.66–1.18
University	0.9	0.73–1.21	1.0	0.69–1.31
Place of residence				
Another place	1		1	
City	1.2	0.99–1.49	1.3*	**1.01–1.65**
Family status				
Unmarried	1		1	
Married	1.2	0.91–1.58	0.9	0.64–1.26
Body mass index				
≥ 25 - < 30 kg/m^2^	1		1	
≥ 30 kg/m^2^	2.3**	**1.90–2.84**	2.8**	**2.17–3.60**
Number of chronic conditions				
No	1		1	
1	2.5**	**1.85–3.38**	1.7*	**1.17–2.56**
2	5.2**	**3.72–7.20**	2.0*	**1.29–3.06**
≥ 3	7.6**	**5.35–11.03**	4.5**	**2.92–7.04**
Number of the visits to a GP per year				
1–2	1		1	
≥ 3	2.6**	**2.03–3.32**	3.2**	**2.23–4.64**
Study years ^a^	1.1**	**1.07–1.15**	1.1*	**1.02–1.13**

^a^Change in odds ratio between two adjacent surveys; statistically significant OR in bold; * - *p* < 0.05; ** - *p* < 0.001;

Abbreviations: *CI* confidence interval, *GP* general practitioner

There were no significant time trends in reported counselling about nutrition and physical activity in women (Table 4).

**Table 4:** Odds ratios of receiving advice from a general practitioner by analysed variables in women with overweight and obesity (multivariate logistic regression analysis)

Variables	Advice on nutrition		Advice on physical activity	
	OR	95% CI	OR	95% CI
Age				
20–34	1		1	
35–44	1.3	0.89–1.80	1.5	0.94–2.44
45–54	1.8*	**1.27–2.42**	1.3	0.83–2.07
55–64	2.2**	**1.54–2.99**	1.8*	**1.17–2.90**
Highest educational attainment				
Secondary school or lower	1		1	
College or Vocational school	0.9	0.78–1.12	1.2	0.94–1.49
University	1.0	0.79–1.21	1.4*	**1.05–1.80**
Place of residence				
Another place	1		1	
City	1.2	1.00–1.37	1.4*	**1.17–1.77**
Family status				
Unmarried	1		1	
Married	1.1	0.92–1.31	0.84	0.68–1.04
Body mass index				
≥ 25 - < 30 kg/m^2^	1		1	
≥ 30 kg/m^2^	2.3**	**1.94–2.68**	2.7**	**2.17–3.29**
Number of chronic conditions				
No	1		1	
1	1.5*	**1.15–1.84**	1.4	0.98–1.86
2	2.4**	**1.89–3.14**	1.9**	**1.34–2.65**
≥ 3	3.4**	**2.57–4.40**	2.6**	**1.84–3.69**
Number of the visits to a GP per year				
1–2	1		1	
≥ 3	2.2**	**1.70–2.81**	1.9**	**1.33–2.64**
Study years ^a^	1.02	0.99–1.06	1.01	0.97–1.05

^a^Change in odds ratio between two adjacent surveys; statistically significant OR in bold; * - *p* < 0.05; ** - *p* < 0.001;

Abbreviations: *CI* confidence interval, *GP* general practitioner

Analysis of pooled data from all surveys showed that the odds of receiving advice on physical activity were higher in men living in cities than in other places (Table 3). Obese men were more likely to report being advised on nutrition and physical activity compared to individuals with overweight. The odds of receiving advice rose with the increasing number of treated or diagnosed chronic conditions as well as with the increasing number of visits to a GP during the last year.

The likelihood of reporting advice on nutrition or physical activity was higher in older than younger women (Table 4). Highly educated women and those living in cities more often reported counselling on physical activity as compared to low educated women and residents of other areas. Obese women were more likely to receive advice from a GP than overweight women. The number of chronic conditions and visits to a GP was positively related to reporting advice.

A reduction in the consumption of fat and sugar and the increase in consumption of vegetables were the most common changes during the last year reported by individuals with overweight and obesity (Table 5).

**Table 5:** The proportion of respondents who reported changes in health behaviours during the last year and odds ratios (OR) for changes by general practitioner advice (advised versus non-advised)

Change in health behaviour	Males			Females		
	percentage in advised respondents	OR*	95% CI	percentage in advised respondents	OR*	95% CI
Reduced use of fat ^a^	52.3	3.4	2.84–4.01	60.5	2.6	2.26–3.05
Changed type of fat ^a^	10.6	2.5	1.79–3.47	9.7	1.8	1.37–2.36
Increased use of vegetables ^a^	39.0	2.0	1.70–2.45	49.9	1.5	1.30–1.74
Reduced use of sugar ^a^	36.7	2.8	2.32–3.44	40.0	1.8	1.57–2.14
Reduced use of salt ^a^	28.8	2.5	2.02–3.07	32.5	2.2	1.82–2.55
Increased physical activity ^b^	26.3	1.7	1.29–2.20	21.7	1.3**	0.99–1.60

^a^by advice on nutrition; ^b^ by advice on physical activity

*p < 0.001 for all ORs, except **p = 0.051;

Abbreviation: *CI* confidence interval

The positive association between being advised and changes in health behaviour was found in both genders. Men and women who received advice from a GP on diet were more likely to report changes in nutrition habits as compared with non-advised individuals. More advised than non-advised men indicated an increase in physical activity during the last year.

## Discussion

Lithuania has undergone major structural changes in the health system after the collapse of the Soviet Union. The primary health care institution was newly established in Lithuania in 1992. Family physicians were retrained from district doctors (paediatricians and internists) or graduated family medicine residency [17]. GP training programme integrates topics on health promotion and disease prevention including weight management. However, our data showed a low rate of counselling of patients with overweight and obesity. Only about a third of respondents reported they received advice on nutrition, even fewer reported advice on physical activity from their GP in the past 12 months. The rate of GP counselling slightly increased from 2000 to 2010; however, we observed a decline in reported advice in the last surveys (2012 and 2014).

Our data are in line with previous studies showing that GPs are not very active in diet and physical activity counselling of their overweight patients [12, 21–23]. In the Netherlands, no significant difference in discussing nutrition during GP consultation was found in the years 1975–2007/2008, while advice on physical activity increased somewhat [24]. A decline in lifestyle counselling for weight reduction was reported in a USA study [25, 26]. A decrease in the prevalence of physical activity counselling was found in Germany [27].

Different barriers to offering lifestyle advice to overweight individuals were reported by GPs. Lack of time, lack of knowledge and poor counselling skills, an insufficient reimbursement for providing counselling, lack of patients’ motivation and lack of administrative support were mentioned as the main barriers that prevent GPs from giving advice [13, 14, 28–30].

Successful intervention and follow-up require time to talk with patients. Because of short patient visit time, comprehensive lifestyle recommendations may not be feasible in practice [31]. Multidisciplinary teams including nurses, dieticians or lifestyle specialists could be involved in the counselling of overweight patients about health behaviour changes.

Good GP knowledge and counselling skills are essential for effective consultation. Providing sufficient training in medical schools and in continuous medical education on how to discuss nutrition and physical activity for weight management and developing the practical tools to support counselling in primary health care may help to increase the frequency of giving advice [8, 32].

Our study showed that GP advice to patients with overweight depended upon patient’s socio-demographic characteristics. We found that the likelihood of reporting GP advice was higher in respondents living in major cities. In Lithuania, cities have an adequate number of GPs’, while rural regions suffer from physician shortage; therefore, city inhabitants have better access to health care [33]. Older and highly educated women were also more likely to be advised. In a Swedish study, men, younger and highly educated patients were more often counselled by GP [12]. Other authors showed that a higher proportion of women and older people received advice [23]. The study carried out in the USA found that disparities in advice to lose weight increased from 1994 to 2000, because a declining trend in counselling was observed among low educated and having low-income population groups, while the prevalence of advice among obese persons with a higher education or in the highest income group remained stable [34]. One of the explanations of such disparities might be that people from certain socio-demographic groups visit their GP more frequently, thus have increased the opportunity to receive weight loss advice. Our data confirm that frequent visits to GP were associated with a higher likelihood of counselling. Older people visit their GP frequently because of health problems. More contacts increase the opportunity for GP to discuss with patients their unhealthy behaviour. Moreover, GPs may consider it is less probable that some people, for example, low educated ones, would accept lifestyle advice and, therefore, they do not discuss change of health behaviour issues with such patients [35].

Consistent with findings from previous studies [21, 36, 37], Lithuanians with higher BMI were more likely to receive advice on nutrition or physical activity from their GP. It is well known that obesity is associated with different health problems, so obese people have a greater chance to visit their GP and discuss with them health behaviour issues. GPs more often have therapeutic than a preventive approach to weight control. They identified weight management as part of chronic diseases care [11]. Patients with therapeutic needs had a higher possibility of being advised to change their diet and to increase their physical activity [21].

Our results suggest that patients who had been counselled by GP on diet and physical activity more often reported lifestyle change: reduced and changed the quality of fat, increased use of vegetables, reduced use of sugar and salt, and increased physical activity.

Previous studies demonstrated that lifestyle advice given by GP can contribute to lifestyle changes and weight loss [12, 16]. A meta-analysis of 12 studies showed that GP advice increased the likelihood of a patient attempting to reduce weight [15]. However, it is difficult to compare different studies because it is not clear to what extent GPs guide their patients on nutrition or physical activity in their busy practice. Some studies mentioned that advice on nutrition and physical activity were quite general without providing detailed strategies [29, 38]. Other studies reported GPs advised on particular nutrients and food products or on eating behaviours [30, 39, 40]. Nevertheless, most authors agreed that the provision of even brief advice might play an important role in changing health behaviours and weight loss. Reduction in body weight shows a positive effect on metabolic health. Even 5% of body weight loss was associated with improvement in the function of insulin-secreting beta cells and insulin sensitivity in fat tissue, liver and skeletal muscle tissue [41]. Modest weight loss significantly improved cardiovascular risk factors (systolic blood pressure, high-density lipoprotein cholesterol and triglycerides), but larger weight losses had greater health benefits [42].

The strengths of this study are as follows. First, the trend analysis was performed using nationally representative data for 14 years. Second, the same questions on reporting GP advice were used in all surveys that allowed comparing the data between the cross-sectional surveys and pooling data for analysis. Third, multiple socio-demographic and other factors were assessed enabling us to identify determinants of GP advice.

Several limitations of our study should be considered. All data were self-reported. A comparison of measured versus self-reported anthropometric data showed a tendency for self-reported height to be overestimated and weight to be underestimated [43]. This can lead to underestimation of overweight prevalence. However, the previous study did not identify a significant difference in the prevalence of obesity defined using self-reported and measured data in Lithuania [44]. Accuracy in the recall of lifestyle advice depended on the memory of participants. However, if a patient does not recall receiving advice, possibly it did not affect their health behaviour. Next limitation is decreasing response rates, especially in the last surveys. Non-response is often associated with unhealthy behaviours and worse health status; therefore, it is possible that our results showed a lower prevalence of overweight and GP counselling on a healthy lifestyle. Moreover, we did not examine the content and quality of GP advice on nutrition and physical activity. More qualified advice may have a better effect on changing patients’ behaviour. Future research is needed to examine the effectiveness of the given advice.

The patients were asked whether they had received advice in the previous year. It is possible that some patients with long overweight and obesity history have been advised many times in the past, but not in the previous year. GP may stop providing advice if patients do not make any efforts to change their lifestyle. Furthermore, we were unable to capture participants who succeeded to change their nutrition habits and physical activity and did not need more GP advice. However, we assume that patients with overweight and obesity should be followed-up by GP to ensure long-term behaviour change through continued motivational support. The study by Hartman SJ et al. showed that enhanced longitudinal intervention with multiple tools such as printed materials and telephone calls, with limited face-to-face counselling, was effective for encouraging weight loss, increasing physical activity and healthy eating [45].

Finally, our data lack information about GP characteristics. It is known that GP socio-demographic data and physical characteristics (being overweight) may influence patients’ perceptions of advice received during counselling and affect a patient’s willingness to follow health advice provided by GP. If a GP is perceived as healthy, the advice that patients receive is generally considered to be more motivating and trustworthy [46]. Unfortunately, our study was anonymous, thus we did not have any possibility to relate the respondents with their GP.

Despite the mentioned limitations, this study offers an overview of how lifestyle counselling is performed in primary health care in Lithuania and assesses the importance of weight loss advice for health behaviour changes. In the period of the growing obesity epidemic, GP can empower their patients to control weight and prevent many diseases by encouraging them to change unhealthy diet and low physical activity.

## Conclusions

The low rate of counselling of patients with overweight and obesity by their GP and significant impact of such counselling on patient attempts to change health behaviours related to weight control highlight the importance of identifying and addressing barriers to advise. Policymakers should recognize the GP needs for appropriate consultation time and additional training in counselling patients on a healthy lifestyle.

## Data Availability

The survey datasets analysed are available from the corresponding author on reasonable request.

## Abbreviations

BMI: Body mass index
CI: Confidence interval
GP: General practitioner
WHO: World Health Organization
